# Diabetic Polyneuropathy Is Associated With Pathomorphological Changes in Human Dorsal Root Ganglia: A Study Using 3T MR Neurography

**DOI:** 10.3389/fnins.2020.570744

**Published:** 2020-09-25

**Authors:** Johann M. E. Jende, Zoltan Kender, Christian Rother, Lucia Alvarez-Ramos, Jan B. Groener, Mirko Pham, Jakob Morgenstern, Dimitrios Oikonomou, Artur Hahn, Alexander Juerchott, Jennifer Kollmer, Sabine Heiland, Stefan Kopf, Peter P. Nawroth, Martin Bendszus, Felix T. Kurz

**Affiliations:** ^1^Department of Neuroradiology, Heidelberg University Hospital, Heidelberg, Germany; ^2^Department of Endocrinology, Diabetology and Clinical Chemistry (Internal Medicine 1), Heidelberg University Hospital, Heidelberg, Germany; ^3^German Center of Diabetes Research, München-Neuherberg, Germany; ^4^Medicover Neuroendokrinologie, Munich, Germany; ^5^Department of Neuroradiology, Würzburg University Hospital, Würzburg, Germany; ^6^Division of Experimental Radiology, Department of Neuroradiology, Heidelberg University Hospital, Heidelberg, Germany; ^7^Joint Institute for Diabetes and Cancer at Helmholtz-Zentrum Munich and Heidelberg University, Heidelberg, Germany

**Keywords:** diabetic polyneuropathy, dorsal root ganglion, magnetic resonance neurography, neuropathic pain, peripheral nervous system

## Abstract

Diabetic neuropathy (DPN) is one of the most severe and yet most poorly understood complications of diabetes mellitus. *In vivo* imaging of dorsal root ganglia (DRG), a key structure for the understanding of DPN, has been restricted to animal studies. These have shown a correlation of decreased DRG volume with neuropathic symptom severity. Our objective was to investigate correlations of DRG morphology and signal characteristics at 3 Tesla (3T) magnetic resonance neurography (MRN) with clinical and serological data in diabetic patients with and without DPN. In this cross-sectional study, participants underwent 3T MRN of both L5 DRG using an isotropic 3D T2-weighted, fat-suppressed sequence with subsequent segmentation of DRG volume and analysis of normalized signal properties. Overall, 55 diabetes patients (66 ± 9 years; 32 men; 30 with DPN) took part in this study. DRG volume was smaller in patients with severe DPN when compared to patients with mild or moderate DPN (134.7 ± 21.86 vs 170.1 ± 49.22; *p* = 0.040). In DPN patients, DRG volume was negatively correlated with the neuropathy disability score (*r* = −0.43; 95%CI = −0.66 to −0.14; *p* = 0.02), a measure of neuropathy severity. DRG volume showed negative correlations with triglycerides (*r* = −0.40; 95%CI = −0.57 to −0.19; *p* = 0.006), and LDL cholesterol (*r* = −0.33; 95%CI = −0.51 to −0.11; *p* = 0.04). There was a strong positive correlation of normalized MR signal intensity (SI) with the neuropathy symptom score in the subgroup of patients with painful DPN (*r* = 0.80; 95%CI = 0.46 to 0.93; *p* = 0.005). DRG SI was positively correlated with HbA1c levels (*r* = 0.30; 95%CI = 0.09 to 0.50; *p* = 0.03) and the triglyceride/HDL ratio (*r* = 0.40; 95%CI = 0.19 to 0.57; *p* = 0.007). In this first *in vivo* study, we found DRG morphological degeneration and signal increase in correlation with neuropathy severity. This elucidates the potential importance of MR-based DRG assessments in studying structural and functional changes in DPN.

## Introduction

Distal symmetric diabetic polyneuropathy (DPN) is one of the most frequent and most severe complications of diabetes mellitus (Papanas and Ziegler, 2015; Nawroth et al., 2017). Although several cellular mechanisms and clinical risk factors associated with DPN have been described, the underlying pathophysiology remains poorly understood (Feldman et al., 2017). One of the major challenges for the investigation of structural changes in the central and the peripheral nervous system in human DPN is that tissue biopsies are mostly restricted to distal nerves like the sural nerve or intradermal nerve fibers (Mohseni et al., 2017). Studies that combine histological analyses of proximal structures like the sciatic nerve or dorsal root ganglia with serological parameters or behavioral traits therefore remain restricted to animal models (Novak et al., 2015).

High resolution magnetic resonance neurography (MRN) at 3 Tesla (3T), however, is a non-invasive technique that allows the detection and quantification of structural nerve lesions in patients at a fascicular level (Jende et al., 2018b, 2019a; Kurz et al., 2018). Recent MRN studies have found that nerve damage in DPN predominates at a proximal level, that proximal nerve lesions are reliably correlated with clinical parameters and serological risk factors, and that structural remodeling of sciatic nerve fascicles differs between painful and painless DPN (Pham et al., 2011; Jende et al., 2018a, 2019a; Groener et al., 2019). The finding of a proximal predominance of nerve lesions in DPN raises the question whether dorsal root ganglia also show structural alterations in DPN (Kobayashi and Zochodne, 2018).

Previous histological studies on the dorsal root ganglion (DRG) of deceased diabetes patients have found morphological changes like thickening of the perineural cell basement membrane, indicating that structural changes in DPN are not restricted to the distal peripheral nerves but also affect the DRG (Johnson, 1983). In addition, it is known from animal studies in streptozotocin (STZ) induced DPN that several metabolic and immunologic processes in the DRG are of importance in painful DPN and that a decrease in DRG volume is associated with the severity of neuropathic symptoms (Sidenius and Jakobsen, 1980; Warzok et al., 1987; Novak et al., 2015; Sango et al., 2017; Kobayashi and Zochodne, 2018). The aim of this study was to investigate correlations between DRG size and normalized signal intensity (SI) in 3T MRN with clinical and serological data in diabetes patients with and without DPN.

## Materials and Methods

### Study Participants

**FIGURE 1:**
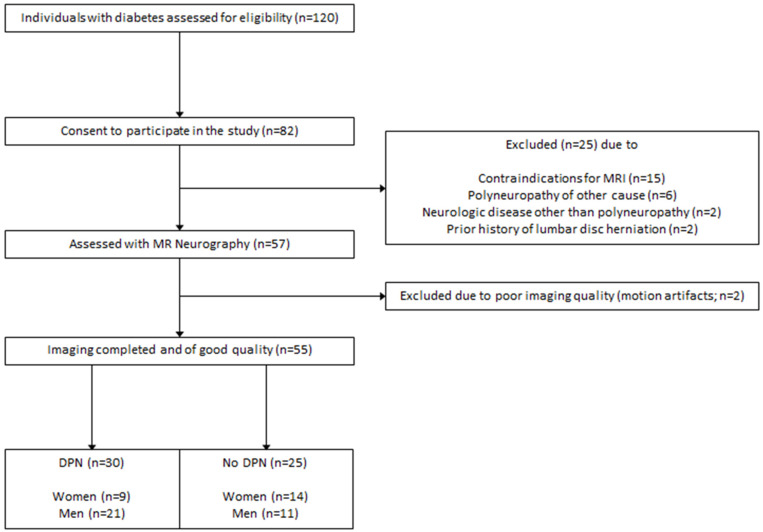
Process of patient recruitment and data acquisition.

This study was approved by the local ethics committee (HEIST-DiC, local ethics number S-383/2016, clinicaltrials.gov identifier NCT03022721). Participants with either type 1 diabetes or type 2 diabetes took part in this prospective, cross-sectional study between September 2016 and June 2018. 120 diabetes patients were approached, of whom 65 were excluded. The process of patient selection is illustrated in Figure 1. Study participants were recruited from the Outpatient Clinic of Internal Medicine of our hospital. Participation in the study was voluntary and all participants gave written informed consent. Overall exclusion criteria were age <18, pregnancy, any contraindications for MRI, any history of lumbar surgery or disc protrusion, any other risk factors for polyneuropathy such as alcoholism, malignant or infectious diseases, hypovitaminosis, monoclonal gammopathy, any previous or ongoing exposure to neurotoxic agents, any chronic neurological diseases such as Parkinson’s disease, restless legs syndrome, or multiple sclerosis.

### Clinical Examination

A detailed medical history was documented and an assessment of neuropathic symptoms was performed in every participant using the neuropathy disability score (NDS) and the neuropathy symptom score (NSS) (Young et al., 1993). In accordance with the guidelines issued by the German Society for Diabetology, the presence of DPN was determined by a score of ≥4 in NDS or NSS. Polyneuropathy was defined as mild to moderate with an NSS <7 or an NDS ≤ 8 and as severe with an NSS ≥ 7 and an NDS > 8. If a discrepancy between NDS and NSS was found, the higher score was chosen (Haslbeck et al., 2004).

Sensory symptoms were derived from the NSS questionnaire. While there are many numerical scales to score pain in DPN, (Shillo et al., 2019) we chose a binary definition for painful and painless DPN depending on whether participants with DPN either experienced painful symptoms for more than 3 months, or not. Painful symptoms were burning, lancinating, or any other painful sensations that could not be explained by other causes than DPN. If participants presented with a combination of painful and painless symptoms (e.g., burning and numbness), DPN was defined as painful.

Blood was drawn in fasting state and proceeded immediately under standardized conditions in the Central Laboratory of our university hospital. The estimated glomerular filtration rate was calculated with the chronic kidney disease epidemiology collaboration formula (Levey et al., 2009).

### MR Neurography Protocol

All participants underwent high-resolution MR neurography of the lumbosacral plexus in a 3T MRI scanner (TIM TRIO, SIEMENS, Erlangen, Germany). The following coils were used: a 32-channel spine coil (Spine 32 3T TIM Coil, SIEMENS Healthineers, Erlangen, Germany) and a 18-channel flex coil (Body 18 3T TIM Coil SIEMENS Healthineers, Erlangen, Germany). MR images were then acquired using a T2−weighted (T2w), three−dimensional inversion recovery sequence with sampling perfection with application−optimized contrasts using different flip angle evolution with the following parameters: field of view = 305 mm × 305 mm, voxel size = 0.95 mm × 0.95 mm × 0.95 mm, variable flip angle variation with (pseudo) steady-state flip angle = 120°, receiver bandwidth = 504 Hz/pixel, repetition time = 3000 ms, echo time = 202 ms, inversion time = 210 ms, echo train length = 209, echo spacing = 14.35 ms, number of signal averages = 2; no parallel imaging, matrix size = 320 × 320 × 104, native acquisition plane: coronal, and acquisition time 8:32 min. Since several studies on DPN in animal models have investigated the L5 DRG and since previous studies on DRG imaging in humans have come to show that L5 DRG are the largest of the lumbar DRG, the sequence was centered to the intervertebral space of L5/S1 (Wattig et al., 1987; Silverstein et al., 2015).

### Image Post-processing

**FIGURE 2:**
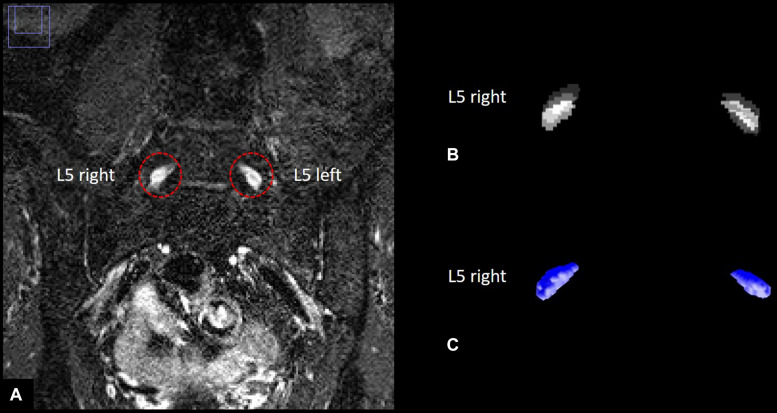
Human dorsal root ganglia (DRG) segmentation. **(A)** Left and right L5 dorsal root ganglion on a T2–weighted, three–dimensional inversion recovery sequence with sampling perfection with application–optimized contrasts using different flip angle evolution. **(B)** Stacks of binarized masks of the left and right L5 dorsal root ganglion. **(C)** Three-dimensional reconstruction of DRG volume.

With 55 participants examined and 104 slices per participant, a total number of 5720 images were acquired. All images were pseudonymized. Images were analyzed in a semi-automatic approach using ImageJ (Rha et al., 2015) and custom-written code in Matlab v7.14.0.0739 (R2012a, Mathworks, Natwick, United States). Anatomical segmentation of both left and right L5 dorsal root ganglion was performed manually for each participant on coronal reformatted images angulated to the intervertebral space between L5 and S1. All images that contained L5 DRG were used for segmentation for each participant. Segmentation was performed manually by two radiologists (JJ and FK) with 5 and 7 years of experience in neuroradiology, respectively, blinded to all clinical data. This produced stacks of binarized images with values 1 for voxels that contained DRG and values 0 for voxels that did not. We used coronal reformats for segmentation since DRG resemble ellipsoids whose main axes form a smaller angle with the normal vector on axial planes than with the normal vector on coronal planes, see e.g., (Hasegawa et al., 1996) that found an angulation of approximately 28 degrees versus the normal vector on axial planes for L5 DRG. DRG voxels at the periphery of each DRG cross-section on every considered plane only contain a part of the respective DRG, thus producing a segmentation error that is proportional to the DRG circumference divided by the DRG area, and which is smaller for segmentation on coronal versus axial reformats due to the typical angulation of L5 DRG and the increased eccentricity of the ellipse-like cross-sections on coronal reformats. The resulting binarized image stacks of L5 DRG were analyzed in Matlab to obtain total DRG volume as the sum of all voxel volumes of both ganglia, respectively. DRG signal intensity values were first standardized to a distribution of signal intensity values of a representative artifact-free adjacent muscle tissue VOI with no discernible crossing vessels by subtracting each DRG signal intensity value with the mean value of the muscle VOI signal intensity distribution and dividing the result with the standard deviation of the muscle VOI signal intensity distribution. The resulting standardized signal intensity values were then normalized by dividing each value with the maximum of the standardized DRG signal intensity values among all participants to obtain units between 0 and 1. An illustration of DRG segmentation and three-dimensional reconstruction of nerve lesions is given in Figure 2.

### Statistical Analysis

Statistical data analysis was performed with GraphPad Prism 6. All data were tested for Gaussian normal distribution using the D’Agostino-Pearson omnibus normality test. If a Gaussian normal distribution was given, *t*-tests were used for comparisons of two groups. If data were not Gaussian distributed, the Mann-Whitney rank sum test was used for comparisons of two groups. Non-parametric Spearman correlation was applied for correlation analysis. All correlations were controlled for age as a potential confounder using partial correlation analysis adjusted for age. For all tests, the level of significance was defined at *p* < 0.05. All results are presented as mean values ± standard deviation (SD). The inter-rater agreement in DRG volume segmentation was determined with the intra-class correlation coefficient (ICC) with the specific model ICC (A,1; McGraw and Wong, 1996). ICC scores below 0.4 are considered as poor agreement, 0.4–0.6 as reasonable agreement, 0.6–0.7 as good agreement, and 0.7–1 as excellent agreement (Bartko, 1991).

## Results

### Demographic and Clinical Data

Overall, 55 participants (mean age 66 ± 9 years, 32 men) with either DPN (*n* = 30) or no DPN (*n* = 25) took part in this study. Six patients were active smokers, whereas 49 patients did not smoke. Of the 30 DPN patients, 19 had mild to moderate symptoms, whereas 11 suffered from severe DPN. Over all participants, NSS and NDS scores were both positively correlated with age (*r* = 0.31; 95%CI = 0.04 to 0.54; *p* = 0.02 and *r* = 0.31; 95%CI = 0.04 to 0.54; *p* = 0.02, respectively). All subsequent correlation analyses were therefore controlled for age as a potential confounder. Of all acquired serological parameters, triglycerides and the triglyceride/HDL index were the only parameters associated with the NDS (*r* = 0.45; 95%CI = 0.25 to 0.62; *p* = 0.001 and *r* = 0.44, 95%CI = 0.24 to 0.61; *p* = 0.003, respectively) and the NSS (*r* = 0.30; 95%CI = 0.08 to 0.49; *p* = 0.04 and *r* = 0.34; 95%CI = 0.12 to 0.52; *p* = 0.03). Triglycerides were higher in DPN patients compared to patients without DPN (236.5 mg/dl ± 248 vs 114.4 mg/dl ± 62.8; *p* = 0.02), whereas the triglycerides/HDL ratio was not (4.95 ± 5.60 vs 2.41 ± 2.16; *p* = 0.27). An overview of clinical, demographic and serological data of study participants is given in Table 1.

**TABLE 1:** Demographic, MRN, and serologic data in patients with and without diabetic neuropathy.

Parameter	DPN (*n* = 30)	No DPN (*n* = 25)	*P*-Value
Women	9	14	n.a.
Men	21	11	n.a.
Type 1 diabetes	8	11	n.a.
Type 2 diabetes	22	14	n.a.
Mean Age (years)	68.38 ± 7.53 (*n* = 31)	59.24 ± 10.08 (*n* = 24)	0.02^M^
Disease duration (years)	24.38 ± 13.56 (*n* = 31)	26.75 ± 13.98 (*n* = 24)	0.53^T^
Body mass index (kg/m^2^)	28.59 ± 4.81 (*n* = 31)	28.09 ± 4.66 (*n* = 24)	0.70^T^
DRG volume (mm^3^)	158.3 ± 44.99	150.2 ± 35.56	0.47^M^
DRG normalized signal intensity	0.524 ± 0.066	0.519 ± 0.114	0.55^M^
HbA1c (mmol/mol) (%)	59 ± 15	59 ± 10	0.54^M^
	7.55 ± 1.38 (*n* = 31)	7.58 ± 0.98 (*n* = 24)	
Creatinine (mg/dl)	0.93 ± 0.24 (*n* = 31)	0.89 ± 0.36 (*n* = 23)	0.26^T^
Glomerular filtration rate (ml/min)	80.69 ± 21.65 (*n* = 29)	80 ± 23.88 (*n* = 24)	0.91^T^
Total cholesterol (mg/dl)	177.60 ± 42.62 (*n* = 29)	176.8 ± 32.86 (*n* = 22)	0.94^T^
LDL cholesterol (mg/dl)	89.51 ± 27.73 (*n* = 28)	96.14 ± 30.75 (*n* = 21)	0.46^T^
HDL cholesterol (mg/dl)	54.65 ± 20.82 (*n* = 27)	57.71 ± 16.55 (*n* = 23)	0.59^T^
Triglycerides (mg/dl)	236.50 ± 248.00 (*n* = 27)	114.4 ± 62.80; (*n* = 22)	0.02^M^
Triglycerides/HDL ratio	4.95 ± 5.60	2.41 ± 2.16	0.27^M^

*All values are shown as mean ± standard deviation. ^*M*^ = *p*-value obtained from a Mann-Whitney test. ^*T*^ = *p*-value obtained from *T*-test. n.a., not applicable; HbA1c, glycated hemoglobin; LDL, low density lipoprotein; HDL, high density lipoprotein.*

### MRN Imaging Data

#### L5 DRG Volume

**FIGURE 3:**
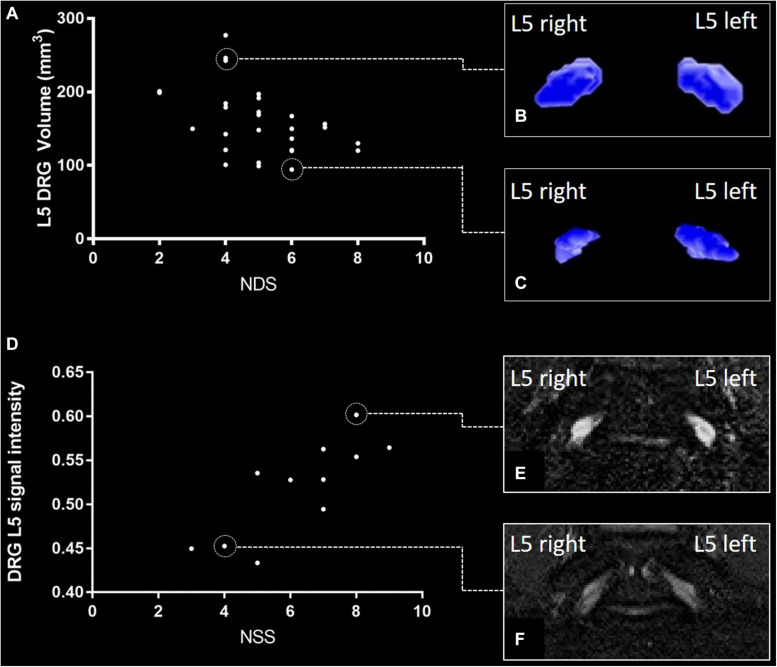
L5 dorsal root ganglion (DRG) volume and normalized signal intensity (SI) versus neuropathy disability score (NDS) and neuropathy symptom score (NSS). **(A)** Correlation of NDS and L5 DRG volume in patients with diabetic polyneuropathy [DPN; (*r* = –0.43; 95%CI = –0.66 to –0.14; *p* = 0.02)]. **(B)** DRG volumetry of a patient with a total L5 DRG volume of 277 mm^3^. **(C)** DRG volumetry of a patient with a total L5 DRG volume of 94 mm^3^. **(D)** Correlation of L5 DRG SI and NSS in painful DPN (*r* = 0.80; 95%CI = 0.46 to 0.93; *p* = 0.005). **(E)** L5 DRG SI in a patient with severe painful DPN (SI = 0.61 ± 0.094). **(F)** L5 DRG SI in a patient with mild painful DPN (SI = 0.45 ± 0.054).

The ICC for DRG volume segmentation was determined as 0.88. DRG volume was negatively associated with NDS (*r* = −0.43; 95%CI = −0.66 to −0.14; *p* = 0.02, Figures 3A–C) in patients with DPN, but not in patients without DPN (*r* = 0.17; 95%CI = −0.26 to 0.54; *p* = 0.43). No correlation was found between DRG volume and the NSS score. In patients without DPN, the only correlation found was between DRG volume and triglycerides (*r* = −0.49; 95%CI = −0.76 to −0.08; *p* = 0.020). No further correlations were found in this group. Although no difference was found between DRG volume in patients with and without DPN (158.3 ± 44.99 vs 150.2 ± 35.56; *p* = 0.47), DRG volume was smaller in patients with severe DPN when compared to mild or moderate DPN (134.7 mm^3^ ± 21.86 vs 170.1 mm^3^ ± 49.22; *p* = 0.04). Patients who were smoking showed smaller DRG volumes than non-smokers (114.0mm^3^ ± 14.36 vs 158.7 mm^3^ ± 40.41; *p* = 0.02). There was no difference in DRG size between painful and painless DPN (149.5 ± 30.02 vs 156.1 ± 43.54; *p* = 0.80). Over all participants, L5 DRG volume showed negative correlations with triglycerides (*r* = −0.40; 95%CI = −0.57 to −0.19; *p* = 0.006), and LDL cholesterol (*r* = −0.33; 95%CI = −0.51 to −0.11; *p* = 0.04). No such correlation was found for disease duration (*r* = −0.12; 95%CI = −0.34 to 0.10; *p* = 0.38), body mass index (*r* = 0.05; 95%CI = −0.17 to 0.27; *p* = 0.72), or HbA1c levels (*r* = −0.17; 95%CI = −0.38 to 0.06; *p* = 0.23).

#### L5 DRG T2-Weighted Normalized Signal Intensity

There was a strong positive correlation of the SI with NSS (*r* = 0.80; 95%CI = 0.46 to 0.93; *p* = 0.005, Figures 3D–F) and a moderate correlation between SI and NDS (*r* = 0.66; 95%CI = 0.21 to 0.88; *p* = 0.04) in patients with painful DPN. No such correlations were found in patients with non-painful DPN (*r* = −0.11; 95%CI = −0.49 to 0.28; *p* = 0.65) or no DPN (*r* = −0.14; 95%CI = −0.52 to 0.29; *p* = 0.50). Over all participants, L5 DRG T2w SI was positively correlated with HbA1c levels (*r* = 0.30; 95%CI = 0.09 to 0.50; *p* = 0.03), triglycerides (*r* = 0.37; 95%CI = 0.16 to 0.55; *p* = 0.01), and the triglycerides/HDL ratio (*r* = 0.40; 95%CI = 0.19 to 0.57; *p* = 0.007). A negative correlation was found between SI and serum HDL (*r* = −0.35; 95%CI = −0.53 to −0.35; *p* = 0.02). No significant correlations were found between disease duration (*r* = −0.08; 95%CI = −0.30 to 0.15; *p* = 0.58), or body mass index (*r* = 0.23; 95%CI = −0.01 to 0.44; *p* = 0.10). No significant difference was found for SI between patients with and without DPN (0.52 ± 0.07 vs 0.52 ± 0.11; *p* = 0.55), between DPN patients with mild or moderate DPN and severe DPN (0.52 ± 0.06 vs 0.53 ± 0.08; *p* = 0.85), or between patients with painful and painless DPN (0.52 ± 0.10 vs 0.52 ± 0.05; *p* = 0.77). An overview of all correlations of DRG imaging parameters with demographic, clinical and serological data is given in in Tables 1–3.

**TABLE 2:** Correlation of dorsal root ganglia volume and normalized signal intensity with clinical parameters.

	L5 dorsal root ganglia volume in mm^3^ (*n* = 55)			L5 dorsal root ganglia normalized signal intensity (*n* = 55)		
	*r*	95%CI	*p*	*R*	95%CI	*p*
L5 dorsal root ganglia volume (mm^3^)	n.a.	n.a.	n.a.	0.12	−0.16 to 0.38	0.39
L5 dorsal root ganglia normalized signal intensity	0.12	−0.16 to 0.38	0.39	n.a.	n.a.	n.a.
NDS (*n* = 55)	<0.01	−0.22 to 0.22	0.99	0.13	−0.10 to 0.34	0.36
NDS DPN (*n* = 30)	–0.43	−0.66 to −0.14	0.02	0.03	−0.28 to 0.34	0.88
NSS (*n* = 55)	0.02	−0.20 to 0.25	0.87	0.10	−0.12 to 0.32	0.46
NSS DPN (*n* = 30)	–0.06	−0.37 to 0.25	0.74	0.22	−0.10 to 0.49	0.28
NSS painful DPN (*n* = 11)	0.33	−0.24 to 0.72	0.36	0.80	0.46 to 0.93	0.005
NSS painless DPN (*n* = 19)	–0.11	−0.49 to 0.28	0.65	–0.09	−0.47 to 0.30	0.71
NDS painful DPN (*n* = 11)	–0.05	−0.56 to 0.49	0.90	0.66	0.21 to 0.88	0.04
NDS painless DPN (*n* = 19)	–0.41	−0.69 to −0.01	0.11	–0.11	−0.49 to 0.28	0.64

*All correlation coefficients are calculated as Spearman coefficients, corrected for age. 95%CI = 95% confidence interval; n.a. = not applicable. NSS = neuropathy symptom score; NDS = neuropathy disability score.*

**TABLE 3:** Correlation of dorsal root ganglia volume and normalized signal intensity with demographic and serologic data.

	L5 dorsal root ganglia volume in mm^3^ (*n* = 55)			L5 dorsal root ganglia normalized signal intensity (*n* = 55)		
	*r*	95%CI	*p*	*R*	95%CI	*p*
BMI (kg/m^2^; *n* = 55)	0.05	−0.17 to 0.27	0.72	0.23	0.01 to 0.44	0.10
Disease duration (years; *n* = 55)	–0.12	−0.34 to 0.10	0.38	–0.08	−0.30 to 0.15	0.58
Creatinine (mg/dl; *n* = 54)	0.06	−0.16 to 0.28	0.66	0.21	−0.01 to 0.42	0.14
Glomerular filtration rate (ml/min; *n* = 53)	0.13	−0.09 to 0.35	0.35	–0.05	−0.27 to 0.18	0.73
Total serum cholesterol (mg/dl; *n* = 51)	–0.25	−0.45 to −0.03	0.08	0.03	−0.19 to 0.25	0.83
LDL (mg/dl; *n* = 49)	–0.33	−0.51 to −0.11	0.04	0.02	−0.20 to 0.25	0.89
HDL (mg/dl; *n* = 50)	0.04	−0.19 to 0.26	0.81	–0.35	−0.53 to -0.35	0.02
Triglycerides (mg/dl; *n* = 49)	–0.40	−0.57 to −0.19	0.006	0.37	0.16 to 0.55	0.01
Triglycerides/HDL ratio	–0.26	−0.46 to −0.04	0.08	0.40	0.19 to 0.57	0.007
HbA1c (mmol/mol; *n* = 55)	–0.17	−0.38 to 0.06	0.23	0.30	0.09 to 0.50	0.03

*All correlation coefficients are calculated as Spearman coefficients, corrected for age. 95%CI = 95% confidence interval; n.a. = not applicable. BMI = body mass index; LDL = low density lipoprotein; HDL = high density lipoprotein; HbA1c = glycated hemoglobin.*

## Discussion

To our knowledge, this MRN pilot study on the L5 DRG in DPN is the first to objectify *in vivo* signs of DRG morphological degeneration in DPN and to investigate whether MR signal alterations of the DRG are correlated with the severity of painful symptoms in DPN. We found that DRG volume was significantly smaller in patients with severe DPN when compared to patients with mild or moderate DPN and, accordingly, that there is a moderate negative correlation between DRG volume and NDS in DPN. We further found a strong positive correlation between DRG SI and NSS in painful DPN. In the context of previous histological studies on DRG in rodent models of DPN, our results indicate that a progression in functional loss in both sensory and motor qualities codified by higher NDS scores is associated with DRG atrophy (Kobayashi and Zochodne, 2018). The structural equivalent for the DRG normalized signal intensity that increases with symptom severity in painful DPN remains to be determined.

The fact that no correlation was found between DRG volume and SI among study participants indicates that changes in DRG SI are not necessarily accompanied by changes in DRG volume and vice versa. This suggests that both parameters are needed to adequately describe DRG changes in T2D DPN with respect to DRG function and clinical DPN severity, thus being two potential indicators for DPN progression.

With regard to serologic parameters, in our cohort, triglycerides were higher in DPN patients when compared to non-DPN patients and showed moderate positive correlations with both NDS scores and DRG SI, while there was a moderate negative correlation with DRG volume. The latter finding may indicate that higher levels of triglycerides are associated with DRG atrophy represented by DRG volume reduction. As the DRG consists of an inner layer comprised of nerve fibers and an outer layer containing the cell bodies of pseudo-unipolar sensory neurons and an adjacent capillary network, it remains to be determined whether a reduction in DRG size in severe DPN is the consequence of damage to one of the two layers or both layers (Sasaki et al., 1997). Since DRG volume was smaller in smokers when compared to non-smokers, and since it is known that smoking causes microvascular damage, it seems likely that damage to the DRG microcirculation is a contributing factor to DRG atrophy in DPN (Tesfaye et al., 2005; Clair et al., 2015). This hypothesis is further supported by the finding of a positive correlation between L5 DRG SI and triglycerides/HDL ratio, since an increase in the latter has been reported to be associated with microvascular pathology (Ain et al., 2019). The correlation of serum triglycerides with both clinical symptom severity and reduced DRG volume is in line with data from clinical studies that have found elevated triglycerides to be a risk factor for nerve damage and increased severity of neuropathic symptoms in DPN. (Tesfaye et al., 2005; Jaiswal et al., 2017). The finding, that both triglycerides and HbA1c levels are associated with an increase in DRG SI, further is in line with findings from a previous MRN study on sciatic nerve lesions in DPN that found T2w-hyperintense nerve lesions to be associated with elevated triglycerides and HbA1c levels (Jende et al., 2019a).

The negative correlation of the DRG volume with triglycerides and the negative correlation of DRG SI with HDL levels are further in line with results from previous MRN studies on the impact of cholesterol levels on sciatic nerve damage in DPN (Andersen et al., 2018; Jende et al., 2019b). The negative correlation between LDL cholesterol and DRG volume, however, contradicts the previous finding that lower LDL cholesterol is associated with sciatic nerve damage in DPN (Jende et al., 2019b). One possible explanation for this discrepancy might be that LDL is required to supply cholesterol to Schwann cells and neurite tips for remyelination after damage to peripheral nerves in DPN, while DRG neurons do not require an equal amount of cholesterol but, instead, as a well vascularized structure, are prone to damage caused by microangiopathy, for which elevated LDL is a risk factor (de Chaves et al., 1997; Vance et al., 2000; Saher et al., 2011; Toth et al., 2012). This assumption, however, remains hypothetical and needs to be investigated by larger longitudinal studies. With regard to the correlation of serum triglycerides with DPN severity and both DRG volume and SI one may argue that triglycerides are elevated in patients with reduced renal function or renal failure and that, accordingly, the correlations found could just be epiphenomena of a reduced renal function in our cohort (Zhang et al., 2019). One has to consider, however, that there was no correlation between GFR or creatinine and triglycerides in our cohort. Still, our data do not allow proving a causal relationship between triglycerides and DRG volume or SI.

Our study is limited by the fact that no electrophysiological recordings were performed on the participants in order to further elucidate the impact of the DRG volume and SI on peripheral nerve function. It is unlikely, however, that changes to an anatomical structure located so far proximally will contribute to detectable and directly attributable changes to peripheral nerves especially at early stages of the disease. Furthermore, nerve conduction studies are limited in localizing disturbances of conduction or sensory action potentials with high spatial accuracy so that point localization to the DRG structure itself remains problematic. Another limitation is that the acquired T2w signal intensity is a non-quantitative parameter that is prone to various potential confounders that can differ between different MRI scanners. One must consider, however, that all images used in this study were acquired at the same scanner and that DRG signal intensity was normalized to adjacent muscle tissue, which should make the results reproducible. In future studies, quantitative T2 imaging of DRG and the assessment of other quantitative MRN imaging parameters such as proton density and fractional anisotropy, that have been shown to be accurate markers of structural peripheral nerve integrity for different neuropathies, should be investigated (Godel et al., 2016; Kollmer et al., 2018; Jende et al., 2019b, 2020; Sollmann et al., 2019; Sato et al., 2020).

The aim of this pilot study was to investigate whether there was a correlation between DRG volume and normalized MR signal intensity of a typical plexus MR sequence, DPN severity and serological risk factors for DPN. We therefore chose the validated scores of NSS and NDS for the assessment of DPN severity. Although all of the risk factors correlated with DRG volume or SI have been shown to be risk factors for the development of DPN in longitudinal clinical studies, (Jaiswal et al., 2017; Andersen et al., 2018) our study does not allow for definite conclusions on a causal relation between serological risk factors and DRG parameters, due to its cross-sectional nature. It should also be considered that the primary aim of this study was to elucidate the use and feasibility of DRG imaging in DPN with regards to DPN severity and serological parameters.

In summary, this study is the first study to image and quantify the DRG in patients with DPN and the first *in vivo* DRG imaging study that found correlations with both clinical parameters of DPN severity and serological data. The study’s findings suggest that DRG volume reduction in DPN is associated with higher levels of triglycerides and that DRG SI, which is associated with symptom severity in painful DPN, is increased by hyperglycemia, and a higher triglyceride/HDL ratio. These results parallel those from peripheral nerve imaging in DPN. Further longitudinal studies are required to investigate the impact of DRG volume and SI on the course of neuropathic symptoms in DPN and to further elucidate the underlying pathophysiological processes.

## Data Availability Statement

The data supporting the conclusions of this article will be made available upon reasonable request by any qualified researcher.

## Ethics Statement

The studies involving human participants were reviewed and approved by Heidelberg University Hospital Ethics Committee. The patients/participants provided their written informed consent to participate in this study.

## Author Contributions

JJ, MP, MB, SH, PN, and FK designed and coordinated the study. JJ, DO, JG, JK, AJ, and FK contributed to the organization of the participants. JJ, CR, MP, AJ, and FK collected the MR data. AH and FK developed image analysis tools. ZK, LA-R, JG, DO, and SK collected clinical, serological and electrophysiological the data. JJ and FK analyzed the data and wrote the manuscript with input from all co-authors. All authors contributed to the article and approved the submitted version.

## Conflict of Interest

MB received grants and personal fees from Codman, Guerbet, Bayer, and Novartis, personal fees from Roche, Teva, Springer, Boehringer, and grants from Siemens. JG was employed by company Medicover GmbH. The remaining authors declare that the research was conducted in the absence of any commercial or financial relationships that could be construed as a potential conflict of interest.

## Abbreviations

DPN: diabetic polyneuropathy
DRG: dorsal root ganglion
HDL: high-density lipoprotein
LDL: low-density lipoprotein
MRI: magnetic resonance imaging
MRN: magnetic resonance neurography
NDS: neuropathy disability score
NSS: neuropathy symptom score
SI: normalized signal intensity
T: Tesla.
